# Phenotypic features of paroxysmal dyskinesia in Dachshund dogs: 62 cases (2017-2025)

**DOI:** 10.1093/jvimsj/aalag195

**Published:** 2026-09-02

**Authors:** Bethany Boyd, Giulia Corsini, Dakir Polidoro, Sofie F M Bhatti, Nicholas Grapes, Diego Alza, Luca Motta, Sara Formoso, Steven De Decker, Simon Bertram, Samantha Gilbert, Athina Karpozilou, Despoina Douralidou, Koen Santifort, João Miguel De Frias, Jos Bongers, Adriana Kaczmarska, Theofanis Liatis

**Affiliations:** Anderson Moores Veterinary Specialists, Linnaeus Veterinary Limited, Mars Veterinary Health, Winchester SO21 2LL, United Kingdom; Anderson Moores Veterinary Specialists, Linnaeus Veterinary Limited, Mars Veterinary Health, Winchester SO21 2LL, United Kingdom; Neurology, AniCura Veterinary Practice Plantijn, 2600 Antwerp, Belgium; AniCura Vetimacs Medical Imaging, 1060 Brussels, Belgium; Faculty of Veterinary Medicine, Ghent University, 9820 Merelbeke, Belgium; Davies Veterinary Specialists, Linnaeus Veterinary Limited, Mars Veterinary Health, Hitchin SG5 3HR, United Kingdom; Veterinary Specialists of Sydney, Miranda 2228 NSW, Australia; Northwest Veterinary Specialists, Linnaeus Veterinary Limited, Mars Veterinary Health, Runcorn WA7 3FW, United Kingdom; Dick White Referrals, Linnaeus Veterinary Limited, Mars Veterinary Health, Newmarket CB8 0UH, United Kingdom; Royal Veterinary College, University of London, Hertfordshire AL9 7TA, United Kingdom; Cave Vet Specialists, Linnaeus Veterinary Limited, Mars Veterinary Health, Wellington TA21 9LE, United Kingdom; Cave Vet Specialists, Linnaeus Veterinary Limited, Mars Veterinary Health, Wellington TA21 9LE, United Kingdom; Southern Counties Veterinary Specialists, Ringwood BH24 3JW, United Kingdom; The Ralph Veterinary Referral Centre, Marlow SL7 1YG, United Kingdom; Evidensia Dierenziekenhuis Arnhem and Hart van Brabant (Waalwijk), 6825 MB Arnhem, The Netherlands; Hospital for Small Animals, Royal (Dick) School of Veterinary Studies, The University of Edinburgh, Edinburgh EH25 9RG, United Kingdom; Moorview Referrals, Newcastle upon Tyne NE23 7RH, United Kingdom; School of Biodiversity, One Health and Veterinary Medicine, University of Glasgow, Glasgow G61 1QH, United Kingdom; School of Biodiversity, One Health and Veterinary Medicine, University of Glasgow, Glasgow G61 1QH, United Kingdom; Plakentia Veterinary Clinic – Alimos, Athens 174 55, Greece; School of Veterinary Medicine, European University Cyprus, Nicosia 1516, Cyprus

**Keywords:** canine, dachshund, dystonia, movement disorder, paroxysmal non-kinesigenic dyskinesia

## Abstract

**Background:**

Paroxysmal dyskinesia (PxD) has not been characterized in Dachshunds.

**Hypothesis/Objectives:**

Describe the phenotype, treatment, and outcome of PxD in Dachshunds.

**Animals:**

Sixty-two Dachshunds with presumptive PxD.

**Methods:**

Observational, retrospective, multicenter study of dogs diagnosed with PxD between 2017 and 2025, with prospective information using an owner questionnaire.

**Results:**

Median age at presentation was 3.2 years (range, 1-11 years). Episodes were characterized by shifting (55/62; 88.7%), single (4/62; 6.5%) limb or cervical dystonia (28/62; 45.2%), tremors (25/62; 40.3%), dystonic head tremors (15/62; 35.5%), truncal sway (20/62, 32.3%), kyphosis (17/62; 27.4%), and ataxia (15/62, 24.2%) or head sway (12/62, 19.4%). Shifting limb dystonia was exhibited in all (19/55; 34.5%), pelvic (7/55; 12.7%), or thoracic (1/55; 1.8 %) limbs. Ptyalism (25/62; 40.3%), vomiting (23/62; 37.1%), or lip smacking (25/62; 40.3%) were common. All dogs were diagnosed with presumptive idiopathic paroxysmal non-kinesigenic dyskinesia. Most common treatments were gluten-free diet (42/62; 67.7%), levetiracetam (9/62; 14.5%), phenobarbital (5/62; 8.1%), or no treatment (18/62; 29%). Follow-up was available for 49 of 62 (79%) dogs with a median follow-up of 543 days (range, 5-2903 days). Twenty-six of 49 (53%) dogs had a decrease, 19 of 49 (38.8%) had no change and 4 of 49 (8.2%) had an increase in the frequency or intensity of episodes.

**Conclusions and clinical importance:**

Paroxysmal dyskinesia occurs in young Dachshunds. Shifting limb dystonia is a common feature, and it can affect the pelvic limbs exclusively, mimicking spinal cord disease. Most dogs experienced a decrease in numbers of events regardless of treatment.

## Introduction

Paroxysmal dyskinesia (PxD) is a movement disorder characterized by recurrent self-limiting episodes of hyperkinetic involuntary movements.^1^ Episode semiology may vary but is generally characterized by dystonia, leading to abnormal posture or gait, and tremors. These episodes may last from seconds to hours.^1^ Three main clinical types of PxD have been described in dogs: paroxysmal kinesigenic dyskinesia (triggered by sudden movement), paroxysmal non-kinesigenic dyskinesia (occurring without a triggering movement), and paroxysmal exercise-induced dyskinesia (triggered by fatigue).^2–4^ Paroxysmal dyskinesia also can be classified according to etiology as genetic, reactive (metabolic, toxic, drug-induced, dietary), structural (secondary to neoplasia, inflammation, infection, or congenital malformation), or of unknown cause (idiopathic).^2^

Paroxysmal dyskinesia in dogs is usually idiopathic and has been reported in several dog breeds^3^ such as Labrador retrievers,^5^ Jack Russell terriers,^5^ and Maltese.^6^

Gluten-free diets have been shown to be effective at decreasing episode frequency and severity both in Border terriers affected by paroxysmal gluten-sensitive dyskinesia (previously called canine epileptoid cramping syndrome),^7^ as well as in a variety of other breeds diagnosed with PxD.^8^ Other reactive causes (eg, PxD associated with insulinoma^9^) are rare in dogs. Structural causes of PxD have been reported rarely in veterinary medicine.^2^ In some breeds, the episodes appear to decrease with age,^5^ but the natural progression of the condition overall is unknown, and different treatments, mainly anti-seizure drugs (eg, phenobarbital, levetiracetam, clonazepam), behavioral drugs (eg, fluoxetine), or acetazolamide, have been tried with variable degrees of success.^2^

To our knowledge, PxD has not been described previously in Dachshund dogs. Our aim was to describe the phenotype, diagnostic findings, treatment, and outcomes of PxD in Dachshund dogs.

## Materials and methods

An observational retrospective multicenter case-series study was conducted using medical records from 14 veterinary hospitals to identify Dachshunds presenting with suspected PxD between 2017 and 2025. Ethical approval was granted by the Royal Veterinary College Social Sciences Research Ethical Review Board (URN SR2024-0158112) and the Veterinary Ethical Review Committee of the Royal (Dick) School of Veterinary Studies (VERC Reference 169.24).

Search terms included “dachshund” and “dyskinesia” or “movement disorder” (or translated equivalents). Inclusion criteria were complete medical records and a presumptive diagnosis of PxD based on video footage or detailed episode descriptions reviewed by a board-certified neurologist or supervised neurology resident. Exclusion criteria included episodes with unclear descriptions or an inconsistent phenotype, cases sharing multiple features with epileptic seizures, myoclonus, idiopathic episodic head tremor, concurrent epileptic seizures, structural brain disease, evidence of clinical progression resulting in persistent neurologic deficits or forebrain signs, and metabolic disorders predisposing to reactive seizures or metabolic encephalopathy.

Complete records included documentation of signalment, medical history including presenting complaints and concurrent diseases, physical and neurologic examination findings, diagnosis, and treatment. Diagnostic investigations, including hematology, serum biochemistry, magnetic resonance imaging (MRI), computed tomography (CT), and cerebrospinal fluid (CSF) analysis were recorded when available (Supplementary Material S4). Video recordings (at presentation or acquired retrospectively) were reviewed when available, but not required for inclusion. When videos were unavailable, owners were shown examples of PxD, focal epileptic seizures, and generalized tonic–clonic seizures to facilitate episode characterization. Episode footage review was used to assess whether events were consistent with PxD, specifically assessing impaired voluntary motor function without tonic–clonic activity, indication of impaired consciousness, or postictal signs, and further characterized the movement phenotype. Clinical examinations and video assessments were performed by a board-certified neurologist or supervised neurology resident. The MRI studies included both high- and low-field systems (Supplementary Material S1).

Outcome data were collected when available. Disease progression was defined as increased episode frequency, intensity, or both. Short-term follow-up was obtained from medical records and communication logs, whereas long-term follow-up was obtained using owner questionnaires. Referring veterinarians were contacted before owners of living dogs were invited by email to complete a consent form and online questionnaire (Supplementary Materials S2 and S3), providing additional information on episode semiology, triggers, disease progression, long-term outcome, and quality of life from the owners’ perspective.

Statistical analyses were performed using SPSS Statistics 26 (IBM Corp., Armonk, NY). Normality was assessed using the Shapiro–Wilk test. Non-normally distributed numerical variables were presented as median, interquartile range (IQR), and range, whereas categorical variables were summarized as counts and percentages. Descriptive statistics were performed.

## Results

Sixty-two dogs from 14 different hospitals across the United Kingdom (*n* = 49), Belgium (*n* = 11), and the Netherlands (*n* = 2) met the inclusion criteria.

### Signalment

Forty-five (72.6%) dogs were male (21 neutered) and 17 of 62 (27.4%) were female (12 spayed). Forty-one (66.1%) dogs were Standard Dachshunds, whereas 21 of 62 (33.9%) dogs were Miniature Dachshunds. Forty-seven (75.8%) dogs were short-haired, 11 of 62 (17.7%) were long-haired and 4 of 62 (6.5%) were wire-haired. Median age at presentation was 3.2 years (range, 1-11 years; IQR, 1.55-4.85 years,) and median body weight at presentation was 5.6 kg (range, 3.8-19.9 kg; IQR, 4.3-6.9 kg,).

### Presenting complaints

All 62 dogs presented for paroxysmal episodes identified as PxD. Median duration between the onset of clinical signs and presentation was 180 days (range, 1-1800 days; IQR, 34.5-325.5 days). Twenty-four (38.7%) owners reported an increase in frequency of episodes between onset and presentation. Upon presentation, 10 of 62 (16.1%) dogs were reported to be anxious, 24 of 62 (38.7%) to have concurrent gastrointestinal signs, and 10 of 62 (16.1%) to have concurrent skin conditions.

### General physical and neurological examinations

On presentation, general physical examination was normal in all dogs, neurologic examination was normal in 61 of 62 dogs (98.4%), with 1 of 62 (1.6%) exhibiting thoracolumbar hyperesthesia that was considered unrelated to the paroxysmal episodes.

### Paroxysmal episode semiology

**Figure 1:**
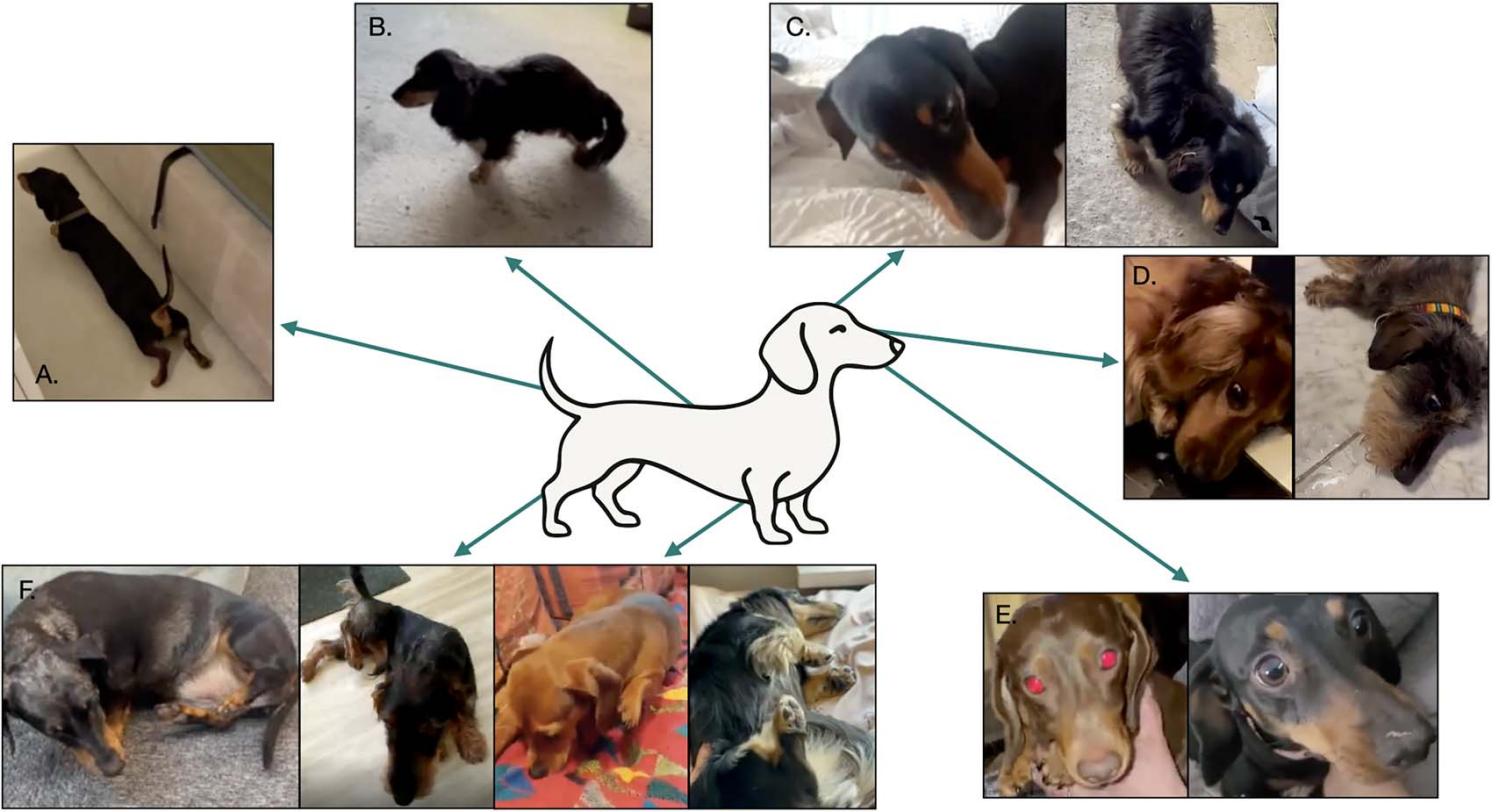
Clinical phenomenology of paroxysmal dyskinesia in Dachshund dogs. (A) tail dystonia and pelvic limb dystonia presented as “pseudoparalysis” of the pelvic limbs; (B) truncal dystonia presented as kyphosis; (C) cervical dystonia presented as head and neck turn; (D) ocular dystonia; (E) bilateral mydriasis during an episode likely reflecting anxiety; (F) limb or shifting limb dystonia presented as “lifting and holding the leg up” at a sustained fashion.

Video footage was available at presentation for neurologist review in 48 of 62 (77.4%) dogs, whereas 30 of 62 (48.4%) were available for secondary analysis at the time of the study. Detailed owner descriptions of the episodes were relied on in 14 of 62 (22.6%) cases. Episode duration varied markedly among dogs and sometimes among episodes in the same dog. Half of the dog population (31/62, 50%) had episodes that lasted between 1 and 5 min, whereas episodes lasting >1 h were reported in 4 of 62 (6.5%), with the longest reported episode lasting 14 h. Episode frequency was also highly variable, and many owners struggled to define how often they occurred. Of the questionnaire responders, 2 of 27 (7.4%) reported multiple episodes each day, 1 of 27 (3.7%) reported them weekly, 7 of 27 (25.9%) monthly, 5 of 27 (18.5%) between 1 and 3 times a year and 12 of 27 (44.4%) could not define a specific pattern in the episode occurrence because it was too variable. Postures adopted during the episodes included lateral recumbency (21/50; 42%), standing (21/50; 42%), and sternal recumbency (8/50; 16%). Kyphosis was present in 17/62 dogs (27.4%). Shifting limb (55/62; 88.7%) or single limb (4/62; 6.5%) dystonia was the most common clinical feature, followed by cervical dystonia (28/62; 45.2%), tremors (25/62; 40.3%), and dystonic head tremors (22/62; 35.5%; Video S1). Shifting limb dystonia included all limbs (19/62 dogs; 30.6%), only pelvic limbs (7/62; 11.3%), or only thoracic limbs (1/62; 1.6%). Other features included truncal sway (20/62; 32.3%), ataxia (15/62; 24.2%), or head sway (12/62;19.4%). Autonomic signs occurring both before and during episodes included ptyalism with or without lip smacking (25/62; 40.3%), vomiting (23/62; 37.1%), and suspected voluntary urination (12/62; 19.4%), defecation (9/62; 14.5%), and mydriasis (8/62, 12.9%) were noticed in some dogs (Figure 1).

### Diagnosis

All dogs were diagnosed with presumptive paroxysmal non-kinesigenic dyskinesia. Magnetic resonance imaging was carried out in 19 of 62 (30.6%) dogs and CSF analysis in 15 of 62 (24.2%) dogs, and both were normal in all cases in which they were evaluated. Reported triggers included stress (10/62; 16.1%), especially when related to startle, sudden movements in the surrounding environment or noises (5/62; 8.1%), dietary changes (6/62; 9.7%), excitement (4/62; 6.5%), administration of medical treatments (2/62; 3.2%), and hormonal fluctuations, such as estrus (1/62; 1.6%).

### Treatment and outcome

Follow-up information was available for 49 of 62 (79.0%) dogs. Median time from presentation to follow-up was 543 days (range, 5-2903 days; IQR, 155-896 days). For these dogs, 26 of 49 (53%) owners reported a decrease in episode frequency, intensity, or both, with 4 of 49 (8.2%) describing a complete cessation of episodes, 19/49 (38.8%) noted no change and 4 of 49 (8.2%) reported an increase in the frequency or intensity of the episodes after diagnosis.

The most prescribed treatment after diagnosis was a gluten-free diet (42/62 dogs; 67.7%). Other treatments included levetiracetam (9/62; 14.5%), phenobarbital (5/62, 8.1%), fluoxetine (3/62, 4.8%), acetazolamide (2/62, 3.2%), vitamin B12 (2/62, 3.2%), imepitoin (1/62, 1.6%), gabapentin, and non-steroidal anti-inflammatory drugs (NSAIDs; 1/62, 1.6%). These treatments had variable success rates (Table 1). Six of 12 (50%) dogs that received no treatment at all and had follow-up available experienced an improvement in their episode frequency, intensity, or both. Three of 34 (8.8%) dogs were changed to a gluten-free diet and their episodes stopped completely, whereas another dog had a good response to the gluten-free diet initially but relapsed 3 years later with more intense episodes. Two of 34 (5.9%) owners, who saw improvement in their dogs’ episodes after the change to a gluten-free diet, expected to see more episodes occur in the days after their dogs scavenged other food. Another dog’s episodes were reported to completely resolve after implementation of lokivetmab (Cytopoint®, Zoetis) treatment for atopic dermatitis. One dog relapsed with more frequent episodes upon discontinuation of levetiracetam treatment, and the drug was restarted.

**Table 1:** Trialed treatments and their effects on episode frequency and/or intensity.

**Trialed treatments**	**No treatment**	**Gluten-free diet**	**Levetiracetam**	**Phenobarbital**	**Fluoxetine**	**Vitamin B12 supplementation**	**Acetazolamide**	**Imepitoin**	**NSAID and gabapentin**
**Decrease in episode frequency and/or intensity**	6/12 (50%)	20/34 (58.8%)	4/8 (50%)	1/4 (25%)	1/2 (50%)	2/2 (100%)	0/1 (0%)	0/1 (0%)	0/1 (0%)
**No improvement**	6/12 (50%)	10/34 (29.4%)	3/8 (37.5%)	2/4 (50%)	1/2 (50%)	0/2 (0%)	1/1 (100%)	1/1 (100%)	1/1 (100%)
**Increasing episode frequency and/or intensity**	0/12 (0%)	4/34 (11.8%)	1/8 (12.5%)	1/4 (25%)	0/2 (0%)	0/2 (0%)	0/1 (0%)	0/1 (0%)	0/1 (0%)
**Total in treatment group with follow-up available**	12 (100%)	34 (100%)	8 (100%)	4 (100%)	2 (100%)	2 (100%)	1 (100%)	1 (100%)	1 (100%)

Twenty dogs were tested for anti-gliadin and anti-transglutami-nase antibodies. Of these, 8 of 20 (40%) were positive for one or the other antibody, 11 of 20 (55%) were negative, and 1 of 20 (5%) had equivocal results for both antibodies. Four of the positive dogs (50%) showed a positive response to implementation of a gluten-free diet, whereas the other 4 of 8 (50%) had no follow-up information available, and only 2 of 11 (18.2%) dogs with negative antibody testing showed improvements with the diet change. Of the remaining nine dogs with negative antibody status, 2 of 11 (18.2%) were lost to follow-up, 1 of 11 (9.1%) showed a worsening of episode intensity, frequency, or both, whereas 6 of 11 (54.5%) showed no change (but 2 of these dogs had too short a follow-up time to assess the clinical relevance of this finding). No follow-up was available for the dog with questionable antibody status.

### Questionnaires

Of the 54 questionnaires sent, 27 of 54 (50%) owners responded. Eleven (40.7%) considered their dogs’ episodes less frequent than before diagnosis, whereas 4 of 27 (14.8%) said that the episodes were more frequent, and 9 of 27 (33.3%) saw no change in episode frequency. When asked about episode intensity, 9 of 27 (33.3%) claimed the episodes were less intense than previously, and 14 of 27 (51.9%) thought the intensity had remained the same, but none noted an increase in episode intensity. Most owners considered the episodes to have little effect on their dogs’ quality of life, with a median grade of 3 of 10 (range, 1-10; IQR, 1-5; where 1 is not at all affected and 10 is severely affected).

## Discussion

Paroxysmal dyskinesia was most frequently found to affect young Dachshunds, with a median age at presentation of 3 years. This observation is consistent with previous studies describing PxD in other breeds, in which mainly young dogs were affected.^10^ In some breeds, including the Labrador retriever and Jack Russell Terrier, clinical signs have been suggested to improve with advancing age.^5^ Given that 50% of dogs with no treatment and available follow-up information in our study showed some degree of improvement, this finding also may be true in Dachshunds. At presentation, 38.7% of Dachshund dogs were reported to have concurrent gastrointestinal signs and 16.1% had concurrent dermatological signs. These signs also have been reported in Border Terriers with paroxysmal gluten-sensitive dyskinesia.^11^ Links between gluten sensitivity, celiac disease, and movement disorders have been described in humans,^12^ with one case report documenting complete resolution of paroxysmal non-kinesigenic dyskinesia symptoms in a child after implementation of a gluten-free diet.^13^

Episode duration and frequency were highly variable among dogs, but they were always self-limiting. Episodes could vary in their appearance among individuals. A small percentage of dogs (8.1%) seemed to have their episodes linked to a startle response with stress or excitement, similar to what had previously been described in Labradors and Jack Russell Terriers.^5^ The owners of 4 dogs (6.5%) mentioned a possible link to sudden movements or exercise, and this small subset may have a disorder more akin to paroxysmal kinesigenic dyskinesia, whereas the majority seem more non-kinesigenic, indicating that there may be different subsets of PxD in this breed and highlighting the difficulties with movement disorder classification in veterinary medicine. The limbs and trunk were the main body areas affected, and the most common clinical finding of PxD in Dachshunds in our study population was shifting limb dystonia characterized by hypertonicity, commonly more severe in the pelvic limbs, with the dog either standing or in lateral recumbency. Cervical dystonia and tremors were also common, along with a truncal or head sway and ataxia. One-third of dogs in our study exhibited a dystonic head tremor during the episodes, which was considered a frequent sign, as previously described in another study.^14^

Dachshunds are well-known to present for neurologic assessment for intervertebral disc disease,^15^ and some of the features of PxD noted in this breed may resemble a spinal disease episode. For example, kyphosis, a posture commonly adopted as a result of thoracolumbar hyperesthesia, was seen in 27.4% of dogs during an episode, likely as part of the truncal dystonia. Additionally, in 7 of 62 (11.3%) Dachshund dogs, PxD only affected the pelvic limbs. In these dogs, owners described that their dogs had an abnormal pelvic limb gait and were struggling with ambulation, as would be the case in dogs with thoracolumbar or lumbosacral myelopathy because of intervertebral disc herniation (IVDH). Moreover, nerve root signature occurs because of pain resulting from entrapment of a nerve root and leads to elevation of the limb and a non-weight-bearing lameness when standing in a dog with intraforaminal IVDH.^16^ Cervical dystonia has also been reported in a Dachshund affected by an extreme lateral intervertebral disc extrusion.^17^ Therefore, IVDH-related clinical signs should be considered a differential diagnosis in dogs exhibiting abnormal limb movements or localized dystonia along with PxD, especially when relying on owner descriptions alone, or in stoic dogs not readily showing discomfort on examination. Knowing how common IVDH is in chondrodystrophic breeds, it is important to always consider other differential diagnoses for the presenting signs. Careful evaluation of the signs, preferably using video footage or witnessing an episode, is of crucial importance.

Focal epileptic seizures often are considered the main differential diagnosis for PxD. Careful observation of an episode, occurring live or based on video footage, by a neurologist often allows differentiation between the 2 conditions. Therefore, episode observation or video analysis is of crucial importance for diagnosis.^1^ However, analysis can be challenging, as described previously^18^ where only a fair level of agreement was observed between veterinary observers asked to classify paroxysmal episodes. Assessment can be especially difficult when considering cases in which more than one type of paroxysmal episode (eg, epileptic seizures and PxD) occurs in a single animal. Electroencephalography (EEG) is the gold standard to differentiate epileptic seizures from other paroxysmal episodes, but limitations concerning the application of this diagnostic tool in dogs and availability in veterinary practices are well known, including the lack of a standardized protocol for acquiring EEG readings in dogs, lack of equipment availability, insufficient cases, and financial cost among factors.^19^^,^^20^ Unfortunately, EEG recordings were not available for any dogs in our cohort, and therefore a type of focal epileptic motor seizure similar to those described previously^21^ cannot be fully ruled out. Generally, PxD is characterized by a normal level of consciousness during the event, lack of a post-ictal phase and autonomic signs, and usually longer episode duration as compared with epileptic seizures.^1^ Differentiation is critical because epileptic seizures may require life-long treatment^22^ and can be life-threatening, whereas PxD typically does not necessarily require any treatment and has a good prognosis.^5^ Nevertheless, this distinction is not always clear-cut, because many Dachshunds exhibited signs both before and during their episodes, in contrast to other breeds such as in the Welsh Terrier.^23^ During our analysis, we found ptyalism in 40.3% of cases, vomiting in 37.1%, urination in 19.4%, defecation in 14.5%, and mydriasis in 12.9%. Some features, such as mydriasis, may be difficult to appreciate on video footage, and it was reported to have a much higher frequency (66.7%) in the owner questionnaires. Mydriasis can occur in dogs that experience anxiety or distress during an episode. In the questionnaire, several owners also reported vomiting as a sign that an episode is imminent. Autonomic signs during PxD episodes have been noted previously in Border Terriers (ptyalism, borborygmi, involuntary urination, and defecation)^24^^,^^25^ and other breeds (ptyalism and vomiting).^6^^,^^14^ Another study excluded 3 Border terriers because they urinated, urinated and defecated, or hypersalivated during their episodes, which were otherwise consistent with PxD.^26^ Autonomic signs observed during PxD episodes could reflect stress-related responses, but autonomic involvement in movement disorders also has been described in humans. Idiopathic cervical dystonia, for example, involves altered sympathetic–parasympathetic regulation related to abnormal neck posture and neurotransmitter alterations,^27^ supporting the possibility that central autonomic circuits may influence the expression of paroxysmal motor events. However, cervical dystonia during an episode did not necessarily coincide with the presence of vomiting, urination, or defecation in our study population. Overactive bladder signs related to dyskinesia episodes have been described in humans affected by Parkinson’s disease. Although the exact mechanisms involved in this interplay are believed to be complex and are not fully understood, it has been hypothesized that disruptions in the basal ganglia responsible for the dyskinesia also may affect inhibitory effects on the voiding reflex.^28^ Therefore, one must be cautious in interpreting the presence of autonomic signs as an indicator of epileptic seizures. Furthermore, when the episodes had a long duration, many dogs were reported to be tired for a while afterwards, which may be confused with abnormal post-ictal behavior. Some owners also notice a behavior change just before the episode, such as seeking reassurance and attempting to reach their owners. A lack of obvious response to interaction during an episode also may be considered by owners to be a loss of consciousness, as reported by 3 of 27 (11%) of our questionnaire responders. Therefore, it is helpful to analyze video footage and take time for focused questioning and history taking, considering all aspects of the episodes when deciding whether the episodes could be epileptic in nature.

Another differential diagnosis that could be considered in Dachshunds presenting with abnormal movements is Lafora disease, which previously has been described in this breed.^29^ A single dog in our study cohort was tested for this disease with a negative result. However, there are several distinguishing factors between these conditions. First, Lafora disease typically is diagnosed in dogs older than those in our cohort, with an average age of onset of 6.94 years.^29^ Second, it is typically characterized by myoclonus,^29^ which represents shock-like movements, similar to the effect seen after stimulating a nerve supplying a muscle,^1^ which we did not see in dogs with suspected PxD. Third, Lafora disease is typically progressive in nature^29^ and lastly, may also include other signs such as generalized seizures, impaired vision, aggression, behavior disinhibition, dementia, and deafness,^29^ none of which were noted in our cohort.

Many drugs have been evaluated for the treatment of PxD, with variable success. Caution is warranted however when interpreting treatment responses, because clinical signs may improve over time independent of treatment, reflecting the natural disease course.^2^^,^^5^ Our results also corroborate this finding, because of the 12 dogs with follow-up that received no treatment, 6 (50%) improved spontaneously. This finding makes it very difficult to interpret treatment efficacy, because improvements may have occurred even without treatment The most commonly suggested treatment was implementation of a gluten-free diet. This approach was recommended in 34 dogs for which follow-up information was available, and 58.8% of these showed improvement in the frequency or intensity or both of their episodes. Improvement in movement disorders after implementation of a strict gluten-free diet has been described in people with evidence of gluten sensitivity.^12^^,^^13^^,^^30^ In dogs, this improvement also has been observed in Border Terriers,^7^^,^^31^ Maltese,^6^ and several other breeds and crossbreeds.^8^ The gluten sensitivity test for diagnosing paroxysmal gluten-sensitive dyskinesia has so far only been validated in Border Terriers,^11^ but it has been used previously in other breeds.^8^ In our cases, a higher percentage of dogs that tested positive for anti-gluten antibodies (either anti-gliadin or anti-transglutaminase) responded to a gluten-free diet compared with antibody-negative dogs. However, there were responders in both groups, and these results indicate that it may be worth considering a diet-trial in affected dogs, regardless of antibody status, as had previously been suggested.^32^ Furthermore, the clinical relevance of these results needs to be considered in light of the small number of cases and lack of follow-up data in a substantial proportion of dogs. Lokivetmab used for atopic dermatitis treatment likely resolved pruritus and stress levels, which subsequently could have triggered a PxD episode.

Given over-representation of the disease in certain breeds, a genetic basis is suspected, and, in a few breeds, a causative genetic mutation has been identified. These include a brevican gene (*BCAN*) mutation in Cavalier King Charles Spaniels,^33^ a *PIGN* gene mutation in Soft-Coated Wheaten Terriers,^34^ a *PCK2* gene mutation in Shetland Sheepdogs,^35^ an *SOD1* mutation in Het Markiesje^36^ and a *TNR* gene mutation in Weimaraners.^37^ Given the lack of any underlying cause identified in those Dachshunds with clinical PxD that underwent investigations, a hereditary etiology should be considered, and a causative mutation may be identified in the future.

Paroxysmal dyskinesia in Dachshund dogs most likely is idiopathic or of genetic origin. Most cases in our study were considered consistent with paroxysmal non-kinesigenic dyskinesias, because they did not appear to be related to sudden movement or exercise, and the most common triggers identified were stress or excitement. Some cases were considered likely to be reactive paroxysmal gluten sensitive dyskinesia, although this etiology could not be proven.

The main limitations of our study include its retrospective nature, and the fact that no EEG examination was available, thus prohibiting the exclusion of an epileptic etiology for these episodes. Phenotyping for PxD is currently underdeveloped in veterinary medicine and therefore presumptive diagnosis is subjective. There was also a possibility for both selection and anchoring bias, because episodes were analyzed and interpreted in light of a presumed diagnosis, which we were also seeking to characterize. Magnetic resonance imaging studies of the brain were also only available in a subset of dogs, and only one MRI and one CT study including the spine were performed, both of which showed evidence of mild disc degeneration. Therefore, intervertebral disc disease with compatible presentations cannot be fully ruled out. Follow-up was also limited, because questionnaires were not available for all dogs, many were lost to follow-up, and the duration of follow-up was variable, potentially affecting interpretation of clinical progression and outcomes. Only small numbers were present in each treatment group, and in some cases, multiple variables were changed simultaneously, and it is challenging to determine which, if any, made a difference. Finally, a subset of dogs did not have a video recording available at the time of presentation and diagnosis was based on narrative descriptions of episodes as well as owner recognition of features in videos of dogs diagnosed with PxD.

In conclusion, PxD in Dachshund dogs in our study was characterized by shifting limb dystonia in most cases, and it sometimes can exclusively affect the pelvic limbs mimicking spinal cord disease.

## Supplementary Material

Video_1_PxD_study_aalag195

Supplementary_Material_aalag195

## Off-label antimicrobial declaration

Authors declare no off-label use of antimicrobials.

## Institutional animal care and use committee or other approval declaration

Ethical approval for the study was granted by the Royal Veterinary College Social Sciences Research Ethical Review Board (URN SR2024—0158112) and the Veterinary Ethical Review Committee of the Royal (Dick) School of Veterinary Studies (VERC Reference 169.24).

## Abbreviations

CSF: cerebrospinal fluid
CT: computed tomography
EEG: electroencephalography
IQR: interquartile range
IVDH: intervertebral disc herniation
MRI: magnetic resonance imaging
PxD: paroxysmal dyskinesia
