# Supplementary material for: Phenotypic features of paroxysmal dyskinesia in Dachshund dogs: 62 cases (2017-2025)
Source: J Vet Intern Med. 2026 Sep 2;40(5):aalag195. doi: 10.1093/jvimsj/aalag195 (PMC13537510; doi:10.1093/jvimsj/aalag195)
Supplement: Supplementary file 1 [file supplementary_material_aalag195.docx]

**SUPPLEMENTARY MATERIAL**

**S1. Details of all MRI devices at different institutions**

- Anderson Moores: 1.5 Tesla Philips Intera (Philips, Best, The Netherlands)
- Davies: 1.5T MRI magnet (Magnetom Sempra, Siemens Healthineers, Erlangen, Germany
- SCVS: Hallmarq 1.5 Small Animal MRI system, Hallmarq Veterinary Imaging, UK
- Cave: Phillips Ingenia 1.5T evolution, UK
- DWR: Hitachi Aperto Lucente 0.4T MRI, Hitachi Medical Corporation, Tokyo, Japan
- Evidensia Small Animal Hospital Arnhem: 1.5 Tesla Canon Vantage Elan (Canon Medical Systems Europe, Amstelveen, The Netherlands)
- UGhent: low-ﬁeld MRI (0.2 T,Airis Mate, Hitachi; or 0.25 T VET MR Grande, Esaote)
- RVC: Intera 1.5T (Running Achieva Software Package), Philips Healthcare, Amsterdam, The Netherlands
- R(D)SVS: Magneton Avanto 1.5 Tesla, Siemens
- Moorview referrals:  1.5 Tesla Canon Medical Systems Vantage Orian MRI scanner
- Glasgow: 1.5 Tesla scanner Siemens Magnetom Essenza; Siemens Healthcare Ltd, Sir William Siemens Square, Frimley, Camberley, Surrey
- North West Veterinary Specialists: 0.4T, Hitachi APERTO Grande, Steinhausen, Switzerland

**S2. Owner consent form**

Paroxysmal dyskinesia (PD) is a type of movement disorder which affects muscle tone and posture. The episodes can last anywhere from seconds to hours, and dogs are usually conscious throughout. Although these episodes do not physically endanger dogs in the same way that seizures may, they can be emotionally distressing for the dogs as well as their owners when they occur. Any dog can be affected by this condition, although it has been specifically described in certain breeds including border terriers, maltese, welsh terriers, scottish terriers and chinook dogs to name a few. We have also noticed that a relatively large number of dachshunds have been seen with these abnormal movements.

For this study looking into paroxysmal dyskinesia in dachshunds, we would like to describe the appearance of the abnormal movement episodes in this breed, as well as see whether we can spot any patterns with regards to potential triggers for the episodes, whether there may be any predisposing factors, and whether there are any treatments or management strategies which may be effective.

You have been contacted since your dachshund has been identified as having been diagnosed with this condition, and we would love to hear from you! If you would like to help us further our understanding of this condition, we would really appreciate it if you could fill in the questionnaire about paroxysmal dyskinesia in Dachshunds. This is comprised of a few general questions about your dog and its medical history, as well as a number of questions looking at the abnormal episodes in more detail, before asking about evolution of the condition since diagnosis and whether any treatment was prescribed and if so, what was the response. At the end of the questionnaire, you will be given the opportunity to upload a video of an episode if you have one. The videos will allow us to analyse the episodes in more detail and get a better understanding of exactly how the dogs act during an abnormal episode. Please note that participation in this study is entirely voluntary, and you do not have to answer any questions that you do not wish to. All data will be processed in accordance with GDPR and data protection regulations.

**S3. Owner questionnaire**

Participation in this questionnaire is completely voluntary and you do not need to answer any questions that you do not wish to. All personal information provided will be kept confidential and held in accordance with applicable data protection legislation and regulations. Any respondents must be 18 years of age or older.

Name of the dog:

Hospital identification number (if known):

Date:

1. What is the sex of your dog?
   a. Male entire
   b. Male neutered
   c. Female entire
   d. Female neutered
2. What is the specific breed of your Dachshund (e.g. longhair, standard, miniature)? (open ended)
3. Does your dog have a pedigree?
   a. Yes
   b. No
4. What is its date of birth? (open ended)
5. Is there any family history of movement disorders?
   a. Yes
   b. No
   c. Not known
6. Do you have video footage of an episode?
   a. Yes
   b. No
7. When the episodes started, was your dog suffering from any other conditions?
   a. Gastrointestinal disturbances (e.g. vomiting, diarrhoea, audible stomach noises, flatulence)
   b. Skin condition
   c. Other (please specify):
8. When did your dog last experience an episode? (open ended)
9. How often do these tend to occur?
   a. Multiple times a day
   b. Once daily
   c. Weekly
   d. Monthly
   e. Other (please describe)
10. Is there a time of day at which they tend to occur more than others?
    a. Morning (6:00-12:00)
    b. Afternoon (12:00-17:00)
    c. Evening (17:00-21:00)
    d. Night (21:00-06:00)
    e. No, they are completely random
    f. Not sure
11. Are you able to predict when your dog will start having an episode?
    a. Yes
    b. No
    c. Not sure
    d. If yes, how do you know?
12. Have you identified any triggers for these episodes?
    a. Yes
    b. No
    c. Not sure
    d. If yes, what are they?
13. Does your dog have any known sensitivity to gluten?
    a. Yes
    b. No
    c. Not sure
    d. If yes, how was it diagnosed?
14. During the episodes, does your dog appear conscious?
    a. Yes
    b. No
    c. Not sure
15. During the episodes, does your dog respond to you either touching or calling them or offering them food?
    a. Yes
    b. No
    c. Not sure
16. During the episodes, does your dog have trouble walking?
    a. Yes
    b. No
    c. Not sure
    d. If yes, please describe how the walking is affected
17. During the episodes, what position does your dog tend to adopt?
    a. Walking
    b. Standing
    c. Sitting
    d. Lying on their side
    e. Lying on their belly
    f. Variable
18. Does your dog show abnormal head movements during the episode?
    a. Yes
    b. No
    c. Not sure
    d. If yes, please describe:
19. Does your dog show abnormal body movements during the episode?
    a. Yes
    b. No
    c. Not sure
    d. If yes, please describe:
20. Does your dog show abnormal leg movements during the episodes?
    a. No
    b. Yes, involving just one leg
    c. Yes, involving the front legs
    d. Yes, involving the back legs
    e. Yes, involving all 4 legs
    f. Not sure
21. Does your dog show any of the following signs during the episodes (please select all that apply)?
    a. Vomiting
    b. Salivation
    c. Defaecation
    d. Urination
    e. Wide pupils
    f. None of the above
22. How long does an episode typically last?
    a. Seconds
    b. 1-5 minutes
    c. 5-10 minutes
    d. 10-30 minutes
    e. 30-60 minutes
    f. Over an hour
    g. Other, please describe:
23. Is your dog normal before and after an episode?
    a. Yes
    b. No
    c. Not sure
    d. If no, please describe:
24. Is your dog normal between episodes?
    a. Yes
    b. No
    c. Not sure
    d. If no, please describe:
25. Is your dog generally anxious or stressed?
    a. Yes
    b. No
    c. Not sure
26. Have you noticed a change in episode frequency since diagnosis?
    a. Yes, they are less frequent than before.
    b. No, the frequency has remained the same.
    c. Yes, they are more frequent than before.
    d. Not sure.
27. Have you noticed a change in episode intensity since starting diagnosis?
    a. Yes, they are less intense than before.
    b. No, the intensity has remained the same.
    c. Yes, they are more intense than before.
    d. Not sure.
28. What diet is your dog currently on? (please be as specific as possible, including food type and brand) (open ended)
29. Has this diet changed since your dog’s diagnosis with paroxysmal dyskinesia?
    a. Yes
    b. No
    c. If yes, please describe how the diet has changed
    d. If yes, have you noticed a change in paroxysmal dyskinesia episode frequency or intensity with the dietary change?
30. Did your dog receive any treatment following the diagnosis of paroxysmal dyskinesia?
    a. Yes
    b. No
    c. If yes, what was prescribed? How long have these treatments been used for? Do you think that this medication has reduced the frequency or intensity of the episodes? Is the dog currently on any treatment for the paroxysmal dyskinesia?
31. Does your dog have any concurrent medical complaints?
    a. No
    b. Yes
    c. If yes, please describe and specify what treatment they are currently on:
32. How affected do you feel that your dog’s quality of life is due to paroxysmal dyskinesia episodes on a scale of 1-10, where 1 = not at all affected and 10 = severely affected?
33. Would you be happy to upload and give consent for research use of a video of your dog’s episode?
    a. Yes
    b. No

Thank you very much for taking the time to answer these questions and helping us increase our understanding of paroxysmal dyskinesias in Dachshunds. It is much appreciated!

**S4 Diagnostic Investigation Results**

| Case | Clinicopathological features | | |  | Imaging | | CSF analysis | Other |
| --- | --- | --- | --- | --- | --- | --- | --- | --- |
|  | Hematology | Biochemistry | Urinalysis | Anti-gluten antibodies | MRI | CT |  |  |
| 1 | Normal | Mildly increased globulin 4.7g/dL (RR 2.5-4.5) |  | Increased |  |  |  |  |
| 2 | Normal | Normal |  |  |  |  |  |  |
| 3 | Normal | ALT mildly increased at 152 U/L (RR 10-125), total amylase slightly decreased 481 nkat/L (RR 500-1500), basal cortisol mildly increased at 132 nmol/L (RR<125nmol/L) |  | Increased: Anti-gliadin IgG 0.32 (RR <0.25), anti-transglutaminase IgA 1.14 (RR <0.41) | Normal |  | Normal |  |
| 4 | Normal | Normal, plasma ammonia concentration was normal (0 µmol/L; RI 0-98 µmol/L). Pre-prandial serum bile acids were mildly increased at 19 µmol/L (RI 0-14.9 µmol/L). |  |  |  |  |  |  |
| 5 | Mild-moderate haemoconcentration with RBC 9.43 x 10^12/L (RR 5.65-8.87), HCT 62.6% (RR 37.3-61.7), HGB 21.4 g/dL (RR 13.1-20.5) | Mildly decreased globulins 19g/L (RR 25-45), ALKP 21 U/L (23-212), fasting ammonia: normal (7 umol/L, ref 0-98), fasting bile acids: normal (<1 umol/L, ref 0-14.9), basal cortisol: not consistent with hypoadrenocorticism (267 nmol/L), vitamin B12: normal (683 ng/L, ref >400) |  | Normal: Anti-gliadin IgG 0.15 (RR <0.25), anti-transglutaminase IgA 0.32 (RR<0.41) |  |  |  |  |
| 6 | Normal | Normal, B12 207pg/mL (RR 275-1000), iCa 1.31 mmol/L (RR1.12 - 1.4) |  | Normal:Anti-gliadin IgG 0.14 (RR<0.25), anti-transglutaminase Ig 0.34 (RR<0.41) |  |  |  |  |
| 7 | Normal | Normal |  |  |  |  |  |  |
| 8 | Normal | Normal |  |  |  |  |  |  |
| 9 | Normal | Normal |  |  |  |  |  |  |
| 10 | Normal | Mild ALT elevation (90 U/l), otherwise normal, resting bile acid normal (6 umol/l) |  |  | Normal |  | Normal |  |
| 11 | Mild leukopenia and mild neutropenia | Normal other than mild elevation in ALT at 113U/L (RR 10-109), mild elevation of cholesterol 10.8 (RR 2.84–8.27 mmol/L) | Normal |  |  |  |  |  |
| 12 | Normal | Normal |  |  |  |  |  |  |
| 13 | Normal | Normal |  |  | Normal |  | Normal |  |
| 14 | Normal | Normal | Normal |  |  |  |  |  |
| 15 | Normal | Normal | Normal |  |  |  |  |  |
| 16 | Normal | Normal | Normal |  |  |  |  |  |
| 17 | Normal | Mild increase in CK at 525 U/L (RR 0-190) |  | Normal |  |  |  |  |
| 18 | Normal | Normal |  | Normal |  |  |  |  |
| 19 | Normal | Normal |  | Increased Antigliadin IgG (1.53 range 0-0.24), Antitransglutaminase IgA unremarkable |  |  |  |  |
| 20 | Normal | Normal |  | Mild increase Antitransglutaminase IgA 0.41 (RR 0-0.40), Antigliadin IgG unremarkable |  |  |  |  |
| 21 | Normal | Normal |  | Increased Antigliadin IgG 0.69 (RR 0-0.24), Antitransglutainase IgA unremarkable |  |  |  |  |
| 22 | Normal | Normal |  | Increased Antitransglutaminase IgA 1.19 (RR 0-0.40), Antigliadin IgG unremarkable |  |  | Normal |  |
| 23 | Normal | Mild elevation in GLDH 29.0 U/L (RR 0 -10 ), mild decrease in cholesterol 3.5 mmol/L (RR 3.8 -7.0 ) |  |  |  |  |  |  |
| 24 |  |  |  |  | Normal brain. There is moderate loss of the normal T2W signal from the nucleus pulposus of the T11-T12 intervertebral disc, as well as some cranial thoracic intervertebral discs. There is a well-defined, focal STIR hyperintensity located within the right dorsal cervical muscles. The other muscular structures visible. |  |  |  |
| 26 | Normal | Normal |  |  | Normal |  | Normal |  |
| 27 | Normal | Normal |  |  |  |  |  |  |
| 28 | Normal | Normal |  |  |  |  |  |  |
| 29 | Normal | Normal |  |  | Normal |  |  |  |
| 30 | Normal | Normal |  |  | Normal |  |  |  |
| 31 | Normal | Normal |  |  | Normal |  |  |  |
| 32 | Normal | Normal |  |  | Normal |  |  |  |
| 33 | Normal | Normal, including T4, TSH and basal cortisol | Normal |  |  |  |  |  |
| 34 | Mild neutrophilia 11.31x10*9 cells/L, (RR 2.5-7.5) | Normal , including bile acid stimulation test | Normal |  | Normal |  | Normal |  |
| 35 | Normal | TLI >50.0ug/L (RR 5.0-32.0), PLI 554ug/L (RR 0-200), otherwise normal, including basal cortisol, B12, folate. | Normal | Normal | Normal |  | Normal |  |
| 36 | Normal | Mild increase in cholesterol 10.5 mmol/L (RR 2.84–8.27) |  |  |  |  |  |  |
| 38 | Normal, including ammonia, bile acid stimulation test, basal cortisol and folate | Normal | Normal, including urine organic acids | Normal | Normal |  | Normal |  |
| 39 | Mild leukopenia | Increased total bilirubin, increased ALT, otherwise normal |  |  | Normal |  | Normal |  |
| 40 | Normal | Normal |  |  |  |  |  |  |
| 41 | Normal | High fructosamine, otherwise normal |  |  |  |  |  |  |
| 42 | Normal | Normal |  |  | Normal |  | Normal |  |
| 43 | Normal | Normal |  | Normal |  |  |  | Echocardiogram: ACVIM stage B1 tricuspid endocardiosis |
| 44 | Normal | Normal |  | Normal |  |  |  |  |
| 45 | Normal | Normal |  |  |  | Mild intervertebral disc disease | Normal |  |
| 46 | Normal | Normal |  | Increased |  |  |  |  |
| 47 | Normal | Normal |  |  | Normal |  | Normal | Negative Lafora disease, normal echocardiogram |
| 48 | Normal | Normal |  |  |  |  |  |  |
| 49 |  |  | Normal |  |  |  |  |  |
| 50 | Mild hemoconcentration | Normal, including bile acid stimulation test, basal cortisol (103nmol/L) and cobalamin (662pg/ml) |  |  |  |  |  |  |
| 52 | Normal | Normal |  |  | Normal |  |  |  |
| 54 | Normal | Normal |  |  |  |  |  |  |
| 56 | Normal | Mild decrease urea 1.9mmol/L (RR 2.5-9.6mmol/L), mild increase triglycerides 1.39mmol/L (RR 0.11-1.13), mild increase AST 58U/L (RR 0-50), otherwise normal, including ammonia, pre-prandial bile acid and basal cortisol |  | Normal |  |  |  |  |
| 57 | Normal | Normal, including B12, folate and basal cortisol |  | Normal |  |  |  |  |
| 58 | Normal | Normal |  | Normal | Normal, incidental partial empty sella syndrome |  | Normal |  |
| 59 | Mild hemoconcentration with Hct 57% (RR 35-55) | Phosphate slightly increased 1.9mmol/L (RR 0.8-1.8). Normal lactate and ammonia |  | Modified Gliadin IgG = positive (1.07; RI: <0.6), and Transglutaminase-2 IgA = questionable (0.67; RI: <0.6). |  |  |  |  |
| 60 | Normal | Normal, including ionised calcium, ammonia and bile acid stimulation test |  | Questionable |  |  |  |  |
| 61 | Normal | Normal, including bile acid stimulation test | Normal |  | Normal |  | Normal | Toxoplasma gondii and Neospora caninum serology negative |
| 62 | Normal | Normal |  |  | Normal |  | Normal, with mild albuminocytological dissociation with normal nucleated cell count (NCC: 3/uL, total microprotein: 42 mg/dL) | Toxoplasma gondii and Neospora caninum serology negative |
